# Toxic epidermal necrolysis

**DOI:** 10.12688/f1000research.7574.1

**Published:** 2016-05-20

**Authors:** Wolfram Hoetzenecker, Tarun Mehra, Ieva Saulite, Martin Glatz, Peter Schmid-Grendelmeier, Emmanuella Guenova, Antonio Cozzio, Lars E. French

**Affiliations:** 1 Department of Dermatology, University Hospital of Zürich, Zürich, Switzerland; 2 Medical Directorate, University Hospital of Zürich, Zürich, Switzerland; 3 Riga Stradins University, Riga, Latvia

**Keywords:** Toxic epidermal necrolysis, skin detachment, keratinocyte cell death, Stevens-Johnson syndrome

## Abstract

Toxic epidermal necrolysis (TEN) is a rare, life-threatening drug-induced skin disease with a mortality rate of approximately 30%. The clinical hallmark of TEN is a marked skin detachment caused by extensive keratinocyte cell death associated with mucosal involvement. The exact pathogenic mechanism of TEN is still uncertain. Recent advances in this field have led to the identification of several factors that might contribute to the induction of excessive apoptosis of keratinocytes. In addition, specific human leukocyte antigen types seem to be associated with certain drugs and the development of TEN. As well-controlled studies are lacking, patients are treated with various immunomodulators (e.g. intravenous immunoglobulin) in addition to the best supportive care.

## Introduction

The exposure to drugs has increased with demographic shifts associated with a higher morbidity of the population. Along with this phenomenon, a rise in the incidence of adverse drug reactions (ADRs) has been observed. Toxic epidermal necrolysis (TEN) is a rare, acute, and life-threatening mucocutaneous disease that is usually drug related. Recent evidence situates TEN as the most severe form amongst a spectrum of severe epidermolytic adverse cutaneous drug reactions, which further include Stevens-Johnson syndrome (SJS) and the SJS-TEN overlap disease ^1^. TEN is a consequence of extensive keratinocyte cell death that results in the separation of significant areas of skin at the dermal-epidermal junction with the production of bullae followed by skin sloughing. This extensive cell death also leads to mucous membrane detachment and contributes to the characteristic symptoms of TEN, which include high fever, mucositis, and moderate to severe skin pain, anxiety, and asthenia. Although the pathogenic mechanism of TEN remains incompletely understood, significant progress in this field of medicine has been made in recent years. The improvements range from the clinical classification that is essential for a better understanding of this disorder to the identification of genetic susceptibilities to certain drugs and the implementation of the first preventive genetic screening measures for selected patient groups and drug classes ^1^. This review aims to provide an up-to-date overview of TEN, emphasizing pathogenesis and immunopathology.

## History and epidemiology

The first description of TEN was made by the Scottish dermatologist Alan Lyell in 1956 ^2^. This severe skin disease, also referred to as Lyell’s syndrome, was initially considered to be a toxic eruption, which closely resembles a severe burn or scalding of the skin ^2^. The skin lesion-associated erythematous plaques and widespread areas of epidermal detachment were referred to by Dr. Lyell as necrolysis. He also described an involvement of the mucous membranes as part of the syndrome and noted that there was very little inflammation in the dermis, a feature that was later referred to as “dermal silence” ^3^. TEN was only associated ^2^ with exposure to certain medications as more patients presenting with TEN were reported subsequent to Lyell’s original publication.

TEN is a rare disease with an annual incidence of approximately 0.4–1.2 cases per million individuals ^4, 5^. There are several factors that seem to impact the incidence of SJS and TEN; regional differences in drug prescription patterns, the population’s genetic background such as human leukocyte antigen (HLA) status and phenotypes of metabolizing enzymes, co-occurrence of cancer, frequency of radiotherapy, and prevalence of certain infectious diseases such as HIV are associated with an increased incidence of TEN ^6, 7^.

## Clinical features

**Figure 1.**
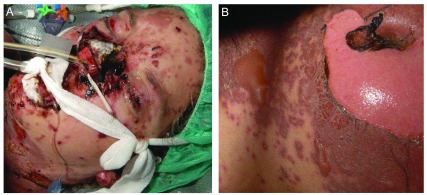
Toxic epidermal necrolysis (TEN) after carbamazepine administration. ( **A**) Skin detachment with facial erosions, including involvement of the lips and conjunctiva. ( **B**) TEN with an extensive cutaneous involvement marked by detached and detachable apoptotic skin erosions on the trunk.

The main symptoms of TEN are usually preceded by non-specific symptoms such as fever, stinging eyes, and discomfort upon swallowing by several hours up to several days. Characteristically, cutaneous lesions first appear in the presternal region as well as the face, palms, and soles of the feet. Mucosal involvement occurs in more than 90% of patients, predominantly affecting the mouth, genitalia, and/or ocular region. In some cases, the respiratory system and gastrointestinal tract are also affected. The morphology of lesions is characterized by erythema and erosions ^8, 9^. Ocular involvement is frequent ^10, 11^. Early cutaneous lesions frequently present as livid, erythematous maculae: they may or may not show signs of slight infiltration. During the course of the disease, the lesions rapidly coalesce and become tense bullae ( Figure 1). With disease progressions, they form large confluent areas of epidermal detachment. The degree of skin involvement is a highly important prognostic factor. Skin involvement should be determined including only already detached necrotic (e.g. blisters or erosions) or detachable skin (Nikolsky positive). A classification system for SJS and TEN according to the extent of skin detachment has been suggested by Bastuji Garin *et al*. ^12^:

- 1–10%: SJS
- 11–30%: SJS-TEN overlap disease
- >30%: TEN

Furthermore, to predict the risk of death in TEN patients, the TEN-specific severity of illness score (SCORTEN) has been proposed ^13^.

SJS and TEN frequently leave cutaneous sequelae after healing. These include cutaneous hyperpigmentation and hypopigmentation (62.5% of cases), nail dystrophy (37.5% of cases), and ocular complications (50% of cases) ^14, 15^.

In most cases of TEN, a strong, direct association of the disease with preceding drug consumption can be established. Indeed, preceding exposure to medications is reported in over 95% of patients with TEN, and a strong association between drug ingestion and cutaneous manifestation is observed in 80% of cases ^1^. Approximately 100 compounds have been identified as the likely triggers of TEN so far, the most frequent being allopurinol, antibiotics, nonsteroidal anti-inflammatory drugs, and anticonvulsants ^1, 16^.

## Pathogenesis

**Figure 2.**
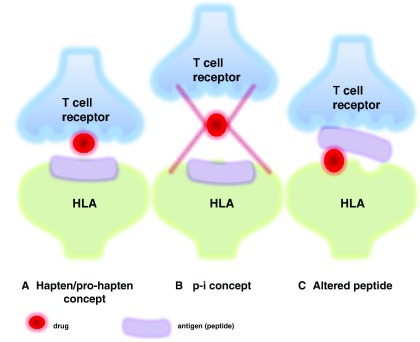
Conceptual models concerning T cell stimulation by drugs in Stevens-Johnson syndrome (SJS)/toxic epidermal necrolysis (TEN). ( **A**) Drugs inducing an adverse skin reaction are not antigenic by themselves. Instead, their immunogenicity may result from binding to carrier proteins, which allows the formation of neoantigens that are recognized by T cells upon presentation by antigen-presenting cells (APCs). ( **B**) The p-i concept is based on the pharmacological interactions of drugs with immune receptors. Consistent with this concept, chemically inert drugs, which are unable to bind covalently to proteins, may activate specific T cells by binding directly to T cell receptors and/or major histocompatibility complex molecules. ( **C**) The association of peptides with HLA molecules is highly specific. According to the “altered peptide model”, specific HLA molecules form a complex with certain drugs, thereby modifying the pool of self-peptides presented to T cells. This may result in increased autoimmunity. Concepts for immunological responses of SJS/TEN modified from Abe *et al*. ^52^.

**Figure 3.**
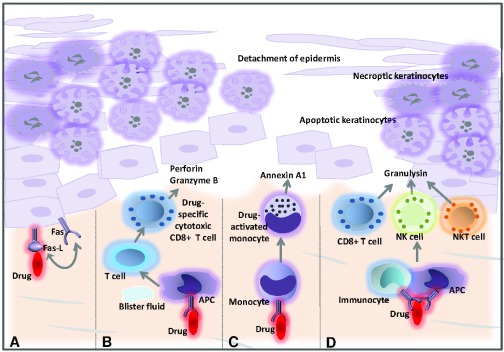
Proposed pathogenic mechanisms in toxic epidermal necrolysis (TEN). ( **A**) The causative medication might induce upregulation of Fas-L by keratinocytes constitutively expressing Fas, leading to activation of a death receptor-mediated apoptotic pathway. ( **B**) The drug might interact with major histocompatibility complex class I-expressing cells, causing drug-specific CD8 ^+^ cytotoxic T cells to accumulate within epidermal blisters, releasing perforin and granzyme B that can kill keratinocytes. ( **C**) Drug-activated monocytes secrete annexin A1, which induces necroptosis in keratinocytes. ( **D**) The drug may also trigger the activation of CD8 ^+^ T cells, NK cells and NKT cells to secrete granulysin, which can induce keratinocyte death without the need for cell contact. This figure has been modified from French *et al.* ^1^. (APC, antigen-presenting cell; NK cell, natural killer cell; NKT cell, natural killer T cell).

So far, the precise molecular and cellular pathogenic mechanisms leading to the development of SJS/TEN can be only partially explained. It is thought to be initiated by an immune response to an antigenic drug-host tissue complex ^9, 17– 20^. Current scientific opinion proposes three different hypotheses as to the formation of the antigenic complex ( Figure 2): i) covalent binding of the drug to a peptide of the cellular surface (hapten/pro-hapten concept); ii) non-covalent, direct interaction of the drug with a specific major histocompatibility complex (MHC) class I allotype (p-i concept); and iii) presentation of an altered-self repertoire by direct drug-MHC I interaction (altered peptide concept). The first, the well-known hapten model, is far less likely to be HLA restricted. However, the two remaining concepts do favor specific HLA phenotypes. According to the latter two hypotheses, a pharmacological agent serving as the allergen would directly bind to specific HLA molecules and/or T cell receptors without prior processing by antigen-presenting cells. In the case of the p-i concept, the mere pharmacological interaction of certain drugs with immune receptors would be sufficient to induce a drug hypersensitivity reaction ^21– 24^. Additionally, recent publications have shown that the HLA-peptide repertoire can be modified by abacavir and carbamazepine, resulting in enhanced peptide presentation and increased autoimmune reactivity (altered peptide model) ^24, 25^. Besides, it has been suggested that SJS may be induced via direct interaction between carbamazepine and HLA-B*1502 ^26, 27^. However, the identification of specific drug-related HLA alleles that strongly increase the likelihood of developing SJS or TEN strengthens the hypothesis of the genetic susceptibility of patients to TEN, supporting the concept of HLA-restricted drug presentation ^28– 30^. This finding is clinically relevant, as screening for the HLA-B*1502 allele in Asian patients prior to drug intake may identify persons at risk of developing severe epidermolytic adverse cutaneous drug reactions, for example in the case of carbamazepine-induced SJS or TEN ^31^. Evidence suggests that immune activation by the drug-host tissue complex induces a strong expression of Fas-L, a cytolytic molecule, on keratinocytes as well as granulysin and annexin A1 secretion by CTLs, NK cells, NKT cells, and monocytes ^32– 37^ ( Figure 3). As a result, Fas-L- and granulysin-mediated apoptosis and/or annexin-dependent necroptosis of keratinocytes with subsequent epidermal necrosis and detachment develop. This indicates that the disturbance of the balance between pro-inflammatory and immunomodulatory mechanisms may critically determine the clinical outcome in cutaneous inflammation. Interestingly, Th17 cells were found alongside CD8 ^+^ T cells in the blister fluid of SJS/TEN patients, but not in patients with erythema multiforme major (EMM). CD8 ^+^ T cells are a source of IL-17, which is a cytokine that promotes the recruitment of neutrophils ^38^. Involvement of skin homing Th17 cells in SJS/TEN is suggested by an observed decrease in the periphery upon treatment-related disease improvement. Recent findings suggest that Th17 cells may alter their phenotype and become regulatory T cells ^39^. Furthermore, recently it has been proposed that Th17 cells originally infiltrate skin lesions in SJS/TEN with regard to the described presence of granulysin-expressing drug-reactive Th17 cells ^40^. The decrease in Th17 cells in patients with resolving SJS/TEN could therefore be associated with a simultaneous rise in regulatory T cells. This hypothesis should be examined in future studies. Neutropenia is generally associated with a higher mortality in SJS/TEN patients ^41^.

## Treatment

Rapid histological examination including direct immunofluorescence analysis of a lesional skin biopsy is paramount in the diagnostic work-up of SJS/TEN, as it helps to rule out diagnoses that can imitate TEN clinically. Autoimmune blistering diseases, fixed drug eruption (FDE), acute generalized exanthematous pustulosis (AGEP), and staphylococcal scalded skin syndrome (SSSS) should be ruled out ( Table 1). The suspect drug should be discontinued immediately and supportive therapy should be ensured in the burn or intensive care unit ^42^. However, valid data on effective therapeutic options are poor, and prospective controlled clinical trials, which can clearly show the benefit of certain treatment options, are lacking. Some case reports and case series report a benefit of cyclosporine, cyclophosphamide, N-acetylcysteine, TNF-α antagonists (e.g. etanercept and infliximab), systemic corticosteroids (favoring pulsed corticosteroid treatment), thalidomide, plasmapheresis, and intravenous immunoglobulin (IVIG) (reviewed in 1). Early administration of high-dose IVIGs (≤2 g/kg) is recommended for patients with TEN, even though its mechanism of action remains unclear ^43– 45^. According to a recent meta-analysis of observational studies, IVIG at dosages of ≤2 g/kg appears to significantly decrease mortality in patients with SJS or TEN ^43^. Alternatively, cyclosporine has shown excellent efficacy for the treatment of TEN in a recent study ^46^. However, as the supporting data for each treatment modality with regard to decreased mortality in TEN are highly controversial, further evidence based on multicenter, randomized, controlled clinical trials is still to be defined.

**Table 1.** Diagnostic algorithm for patients with suspected severe adverse cutaneous drug reactions. AGEP, acute generalized exanthematous pustulosis; DRESS, drug reaction with eosinophilia and systemic reaction; MPR, maculopapular rash; SJS, Stevens-Johnson syndrome; TEN, toxic epidermal necrolysis.

Suspicion of severe adverse drug reaction					
Facial edema Blood eosinophilia Mucous or conjunctival lesions Painful eyes or skin Epidermal detachment/erosions					
MPR	DRESS	AGEP	SJS/TEN		
Maculopapular exanthema No skin detachment No organ involvement	Facial edema Maculopapular exanthema Fever Blood eosinophilia Leukocytosis with atypical lymphocytes Organ involvement (lymph nodes, liver, and kidneys)	Acute sterile pustular eruption Erythema of the skin Fever Blood neutrophilia Facial edema	Painful maculopapular exanthema Possibly atypical targetoid lesions Skin detachment Mucosal and conjunctival erosions		
			SJS	SJS/TEN	TEN
			Detached/ detachable skin <10%	Detached/ detachable skin 10%–30%	Detached/ detachable skin >30%

## Allergologic work-up

The allergologic work-up to identify the causative agents includes skin tests (epicutaneous testing ^47^), *in vitro* assays (lymphocyte transformation tests [LTTs] ^48– 50^), and drug-induced cytokine production assays (e.g. enzyme-linked immunospot [ELISpot] ^51^). Skin tests have been shown to be safe in TEN patients, but their specificity and sensitivity are rather low ^51^. In a recent report, Barbaud *et al*. performed skin patch testing to identify the causative agent in 17 patients who had suffered from SJS and/or TEN. Positive patch test reactions were observed in only 24% of those patients ^52^. Concerning *in vitro* tests, it should be noted that the LTT is not a standardized procedure and merely demonstrates the proliferation of lymphocytes in the presence of various compounds. However, LTT in patients with SJS/TEN has shown low sensitivity, even if performed by highly qualified personnel ^53^.

## Conclusion

Since the time TEN was first described by Dr. Lyell, it has remained a deadly disease with a mortality of around 30%. There is an unmet need to study the pathophysiology of TEN in more detail, which is complicated by the rarity of this disease and the lack of appropriate mouse models. Additionally, effective therapeutic options validated by prospective, randomized, controlled trials remain to be discovered. The most important therapeutic measure so far remains the rapid identification and withdrawal of the causative drug in addition to supportive care. However, this can be a complicated task in patients with polymedication. The allergologic work-up is further complicated by the lack of safe test methods with a high sensitivity and specificity.
